# PYF: a multi-functional algorithm for predicting production and optimizing metabolic engineering strategy in *Escherichia coli* microbial consortia

**DOI:** 10.1093/bib/bbaf295

**Published:** 2025-06-21

**Authors:** Chen Yang, Yingqi Zhao, Boyuan Xue, Shaojie Wang, Haijia Su

**Affiliations:** State Key Laboratory of Green Biomanufacturing, National Energy R&D Center for Biorefinery, Beijing Key Laboratory of Green Chemicals Biomanufacturing, Beijing Synthetic Bio-manufacturing Technology Innovation Center, Beijing University of Chemical Technology, No.15, Beisanhuan East Road, Beijing 100029, PR China; State Key Laboratory of Green Biomanufacturing, National Energy R&D Center for Biorefinery, Beijing Key Laboratory of Green Chemicals Biomanufacturing, Beijing Synthetic Bio-manufacturing Technology Innovation Center, Beijing University of Chemical Technology, No.15, Beisanhuan East Road, Beijing 100029, PR China; State Key Laboratory of Green Biomanufacturing, National Energy R&D Center for Biorefinery, Beijing Key Laboratory of Green Chemicals Biomanufacturing, Beijing Synthetic Bio-manufacturing Technology Innovation Center, Beijing University of Chemical Technology, No.15, Beisanhuan East Road, Beijing 100029, PR China; State Key Laboratory of Green Biomanufacturing, National Energy R&D Center for Biorefinery, Beijing Key Laboratory of Green Chemicals Biomanufacturing, Beijing Synthetic Bio-manufacturing Technology Innovation Center, Beijing University of Chemical Technology, No.15, Beisanhuan East Road, Beijing 100029, PR China; State Key Laboratory of Green Biomanufacturing, National Energy R&D Center for Biorefinery, Beijing Key Laboratory of Green Chemicals Biomanufacturing, Beijing Synthetic Bio-manufacturing Technology Innovation Center, Beijing University of Chemical Technology, No.15, Beisanhuan East Road, Beijing 100029, PR China

**Keywords:** microbial consortium, metabolic simulation algorithm, biosynthesis pathway expression degree, production prediction, metabolic engineering strategy optimization

## Abstract

Simulating production in microbial consortia is crucial for optimizing metabolic engineering strategies to achieve high yields. However, existing algorithms for modeling polymicrobial metabolic fluxes, based on genome-scale metabolic networks, often overlook the conflicts and coordination between biosynthesis tasks and self-growth interests, leading to limited prediction accuracy. This study introduces the Polymicrobial cell factory Yield Forecasting (PYF) algorithm, which simulates the relationships between biosynthesis and growth more effectively by incorporating the expression degrees of biosynthesis pathways. PYF was shown to accurately predict the production of *Escherichia coli*–*E. coli* consortia under various scenarios, including mono-metabolite exchange, dual-carbon sources, and dual-metabolite exchange. The results revealed a mean relative error (MRE) of 0.106, an average determination coefficient of 0.883, and an average hypothesis testing parameter of 0.930 between predicted and experimental productions. Compared with the recent metabolic simulation algorithm, PYF reduced the MRE by ~61.6%. PYF is adaptable and enables accurate simulation even without enzyme catalytic data. Meanwhile, PYF rapidly analyzed and optimized metabolic engineering strategies through sensitivity analysis. By eliminating the need for specialized division and integration of polymicrobial metabolic networks, PYF greatly simplifies the simulation process, offering a novel approach for predicting and enhancing production in microbial consortia.

## Graphical Abstract

**Graphical Abstract:**
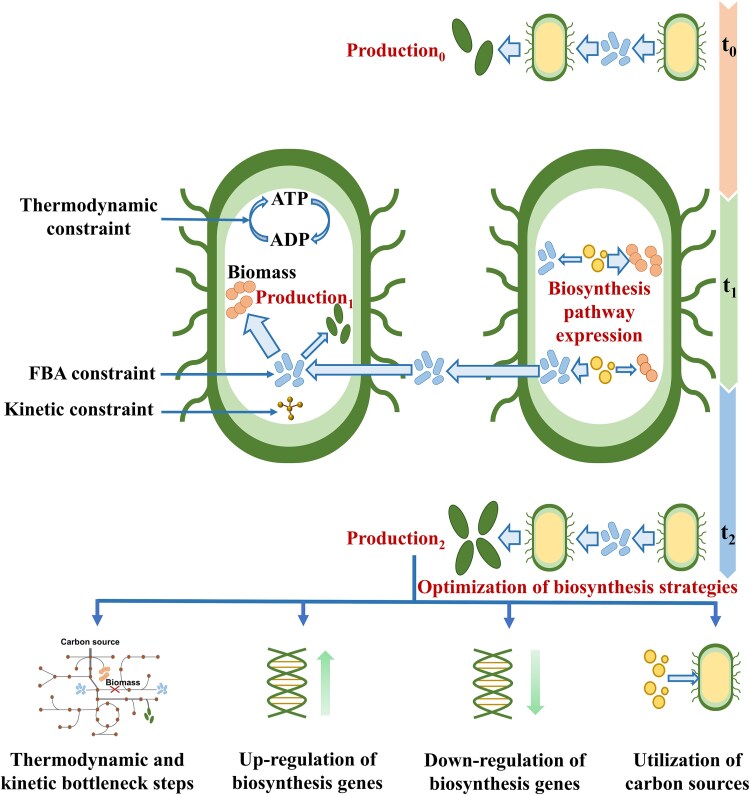

## Introduction

Synthetic microbial consortia (also known as co-culture systems), composed of intentionally designed communities of microorganisms, have gained increasing attention due to their potential in optimizing complex biological processes [1]. By leveraging the complementary functions of different microbial species, these consortia can achieve outcomes that surpass those of individual strains. Key advantages include enhanced metabolic efficiency, greater adaptability to environmental variations, and the ability to perform complex biochemical transformations. These characteristics make synthetic consortia particularly promising for the biosynthesis of high-value biochemicals such as naringenin [2] and genistein [3], biosynthesis of new biochemical not present in mono-cultures [4], and biosynthesis of expensive biochemical without inducers and precursors [5]. However, accurately predicting the productivity of such systems presents a significant challenge. The complex relationships between biosynthesis tasks and growth interests, ranging from conflict to balance, and the complex intracellular environments make it difficult to accurately model metabolism with traditional methods for microbial consortia. As a result, advanced algorithms capable of modeling these intricate relationships and environments are urgently needed to improve prediction accuracy and system optimization rationality.

Genome-scale metabolic networks have provided a large amount of cellular metabolic information such as genome-scale metabolic reactions, enzymes, and genes, promising to model the biosynthesis–growth relationships and complex intracellular environments. For metabolism of microbial consortia, it has been widely applied in the simulation of strain abundance, growth, and interactions with constraints reflecting intracellular environments. For example, under the flux balance analysis (FBA) constraint [6], algorithms based on the “overall optimization” assumption [7] calculate strain abundance and growth rate of microbial consortia with relatively low computational cost [7, 8]. However, “overall optimization” assumes that the overall growth rate is maximized, subordinating individual growth interests to population-level growth, which often yields low accuracy for production predictions and fails to simulate stable metabolic fluxes under competitive conditions.

To address the challenge of polymicrobial metabolism simulation in competitive conditions, algorithms based on the “Nash equilibrium” assumption [9] were developed under the FBA constraint [6, 10]. These algorithms improve upon the “global optimum” approach by allowing individual strains to adjust their metabolic strategies in response to the activities of other strains [10]. This approach better captures the influence of individual interests on population-level outcomes [10]. However, it struggles to accurately predict productions and metabolic fluxes in microbial consortia due to limited consideration of intracellular enzyme environments [11, 12] and thermodynamic environments [12]. Additionally, this approach fails to simulate production and metabolism when a Nash equilibrium solution is unavailable [13].

Recently, a novel algorithm, CulECpy, was introduced to optimize biosynthesis conditions for microbial consortia, including strain ratios and bottleneck reactions [14]. CulECpy has alleviated certain limitations in simulating intracellular enzyme environments by combined FBA [6] and enzyme kinetic constraints [11, 12], proving adaptable and effective in optimizing biosynthesis conditions for products like rosmarinic acid [14, 15], sakuranetin [14, 16], and curcumin [14, 17]. However, as CulECpy primarily focuses on pathway assignments across strains without fully addressing cellular thermodynamic environments, it remains limited in its quantitative production simulations. Moreover, the complexity arising from the global coupling of population- and individual-level metabolic fluxes, as well as the division and integration of polymicrobial metabolic networks, further limits its predictive power.

Importantly, the above simulation algorithms insufficiently consider the biosynthesis–growth relationships, and insufficiently consider the intracellular thermodynamic environments, resulting in low adaptability in metabolic flux simulation and limited accuracy in production simulation. To address these problems in predicting productions of microbial consortia, this study proposes the Polymicrobial cell factory Yield Forecasting (PYF) algorithm based on the expression degree of biosynthesis pathways. PYF models the biosynthesis–growth relationships by exploring pathway expression levels and simulates the intracellular thermodynamic environments by a combination of FBA, enzyme kinetic, and thermodynamic constraints. PYF accurately predicts the biosynthesis efficiencies of different genetically modified *Escherichia coli* consortia under various scenarios, including mono-metabolite exchange, dual-carbon sources, and dual-metabolite exchange. PYF enables rational optimization of metabolic engineering strategies via sensitivity analysis of production simulation results. The codes of PYF are available at GitHub (https://github.com/ziyuanzu/PYF).

## Methods

### Symbols and descriptions

The symbols and descriptions used by the PYF algorithm are listed in Table 1.

**Table 1:** Symbols and descriptions

Symbols	Descriptions
$\overline{v_{bio}^{dec}}$	The growth rate decreased by forcing gene expression
$\overline{v_{bio}^{nfeg}}$	The growth rate without forced expression genes
$\overline{v_{bio}^{feg}}$	The mean growth rate with forced expression genes
$d$	Biosynthesis pathway expression degree
$\overline{v_{bio}}$	The mean growth rate
*B*	The maximum–minimum driving force of the pathway
*i*	The serial number of a reaction
*j*	The serial number of a strain
${Df}_i$	The thermodynamic driving force of the *i*-th reaction
${\Delta }_r{G}_i^{\prime }$	The Gibbs free energy of the *i*-th reaction
${\Delta }_r{G}_i^{\prime 0}$	The standard Gibbs free energy of the *i*-th reaction
*R*	Gas constant
*T*	Temperature
*n*	The number of reactions
${S}_i^T$	The transposed vector in stoichiometric matrix of the *i*-th reaction
*C*	The metabolite concentrations
${v}_i$	The flux of the *i*-th reaction
${UB}_i$	The upper bound of the *i*-th reaction
${z}_i$ and $K$	A binary variable and a sufficiently large value to realize the thermodynamic constraints only for the pathway reactions
${Df}_{min}$	The vector of the minimum thermodynamic driving force
${C}_{min}$ and ${C}_{max}$	The vector of lower and upper bounds of the logarithms of the metabolite concentrations, respectively
${k}_j$	The multiplicity of the instantaneous fluxes corresponding to the mean fluxes for the *j*-th strain
${C}_{initial,j}$	The initial environmental carbon source concentration for the *j*-th strain
$\overline{C_j}$	The average environmental carbon source concentration for the *j*-th strain
${icr}_j$	The inoculum ratio of the *j*-th strain
*k*	The mapping constant of instantaneous fluxes in co-cultures corresponding to the mean fluxes in mono-cultures
${v}_{t,i}$	The instantaneous flux of the *i*-th reaction at the time point *t*
$\overline{v_i}$	The mean flux of the *i*-th reaction
${C}_{t, production}$	The production from the time point *t* to the time point $t+\Delta t$
${v}_{t, production,j}$	The instantaneous flux of product biosynthesis for the *j*-th strain at the time point *t*
$\Delta t$	The time interval between iterations
*T*	Time point
$tp$	The number of time points
${C}_{production}$	The production of a two-strain cell factory
$puf$	The change ratio of the production to the feature
${C}_{production}^{\ast }$	The production after biosynthesis strategy optimization
${x}^{\ast }$	The feature value after biosynthesis strategy optimization
*x*	The initial feature value
$Rpuf$	The change ratio of the production to the unit change of a feature

### Simulation method for relationships between biosynthesis tasks and growth interests based on biosynthesis pathway expression degrees

#### Simulation method for the biosynthesis pathway expression degrees

The metabolic fluxes in mono-cultures were simulated to calculate the maximum productions under different lower bounds of growth rates and different carbon source concentrations. The optimal lower bound of growth rate was then chosen to make the simulated production as close as possible to the measured value. Finally, the biosynthesis pathway expression degree was calculated separately based on the optimal lower bound of the growth rate in a mono-culture, as shown in Eqs. (1)–(2).


(1) \begin{equation*} \overline{v_{bio}^{dec}}=\overline{v_{bio}^{nfeg}}-\overline{v_{bio}^{feg}} \end{equation*}

(2) \begin{equation*} d=\overline{v_{bio}^{dec}}/\overline{v_{bio}^{nfeg}} \end{equation*}

#### Simulation method for metabolic fluxes in mono-cultures

The mean fluxes in mono-cultures for genome-scale metabolism were simulated based on FBA [6], enzyme kinetic [11, 12], and the Max-min Driving Force (MDF) thermodynamic constraints [12, 18]. The optimal planning method constrained the solution space and simulated the mean intracellular metabolic fluxes through the program package of Xue Yang *et al*. [12] and the program package of Pyomo [19]. For a wild strain, the FBA constraint and the kinetic constraint are shown in the literature of Xue Yang *et al*. [12] and a previous study [20], and the MDF thermodynamic constraint is shown in Eqs. (3)–(9).

The target functions


(3) \begin{equation*} {{Maximize}}\ \overline{v_{bio}^{nfeg}} \end{equation*}

(4) \begin{equation*} {\displaystyle \begin{array}{c}{{Maximize}}\\{}C,B\end{array}}\ B \end{equation*}

The thermodynamic constraint


(5) \begin{equation*} {Df}_i=-{\Delta }_r{G}_i^{\prime }=-\left({\Delta }_r{G}_i^{\prime 0}+ RTn{S}_i^T nlnC\right) \end{equation*}

(6) \begin{equation*} \overline{v_i}\le{z}_in{UB}_i \end{equation*}

(7) \begin{equation*} {Df}_i+\left(1-{z}_i\right) nK\ge{Df}_{min}\ge 0 \end{equation*}

(8) \begin{equation*} {Df}_i\ge B \end{equation*}

(9) \begin{equation*} {C}_{min}\le C\le{C}_{max} \end{equation*}

For biosynthesis efficiency simulation with forced expression genes, the mean productions during the culture time can be simulated by changing the main target function and adding a new constraint, as shown in Eqs. (10)–(11).

The changed main target function of mean productions


(10) \begin{equation*} {{Maximize}}\ \overline{v_{production}} \end{equation*}

The added lower bound constraint on mean growth rates


(11) \begin{equation*} \overline{v_{bio}}\ge \overline{v_{bio}^{feg}}=\overline{v_{bio}^{nfeg}}-\overline{v_{bio}^{nfeg}}\cdot d \end{equation*}

### Prediction method for the productions of two-strain consortia

#### Calculation of the mapping constant for mean-instantaneous metabolic fluxes

For a mono *j*-th strain, the multiplicity ${k}_j$ of the instantaneous fluxes corresponding to the mean fluxes was calculated based on the ratio of the initial carbon source concentration to the average carbon source concentration, as shown in Eq. (12). Then, the mapping constant *k* of the instantaneous fluxes in co-cultures corresponding to the mean fluxes in mono-cultures was calculated through the strain inoculation ratio, as shown in Eq. (13).


(12) \begin{equation*} {k}_j={C}_{initial,j}/\overline{C_j} \end{equation*}

(13) \begin{equation*} k=\sum_{j=1}^2{k}_j\cdot{icr}_j \end{equation*}

#### Discrete method for production simulation

According to the calculated mapping constant *k*, the productions during $\Delta t$ in the two-strain cell factory was mapped to the mean fluxes in mono-cultures through a discrete method, as shown in Eqs. (14)–(15). Then, the production of the two-strain cell factory can be predicted through the productions during $\Delta t$, as shown in Eq. (16).


(14) \begin{equation*} {v}_{t,i}=k\cdot \overline{v_i} \end{equation*}

(15) \begin{equation*} {C}_{t, production}=\sum_{j=1}^2{v}_{t, production,j}\cdot \Delta t \end{equation*}

(16) \begin{equation*} {C}_{production}=\sum_{t=1}^{tp}{C}_{t, production} \end{equation*}

### Optimization method of biosynthesis strategies based on sensitivity analysis

The effect of unit change of a feature on the production was calculated through the change ratios of the production to the feature and through the predicted production, as shown in Eqs. (17)–(18).


(17) \begin{equation*} puf=\left({C}_{production}^{\ast }-{C}_{production}\right)/\left({x}^{\ast }-x\right) \end{equation*}

(18) \begin{equation*} Rpuf= puf/{C}_{production} \end{equation*}

## Results and discussion

### Design and characteristics of PYF algorithm for production prediction and biosynthesis strategy optimization of two-strain consortia

Genome-scale metabolic networks play a critical role in enabling accurate production simulations and providing rational guidance for biosynthesis processes. However, traditional algorithms inadequately address the interplay between biosynthesis tasks and self-growth interests and exhibit limited application of multiple constraints, which compromise simulation accuracy. To address these limitations, we developed the PYF algorithm, which incorporates biosynthesis pathway expressions, FBA [6], enzyme kinetic constraint [11, 12], and thermodynamic constraint [12, 18] for accurate production prediction.

**Figure 1:**
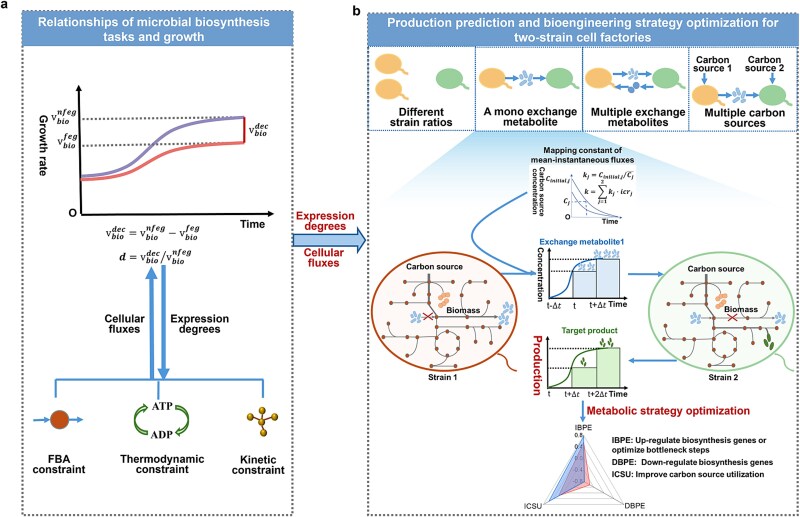
Schematic diagram of the PYF simulation algorithm.

PYF improves the simulation accuracies of metabolic fluxes of wild strains in mono-cultures by employing FBA [6], enzyme kinetic constraint [11, 12], and thermodynamic constraint [12, 18]. As shown in Fig. 1a, the algorithm simulates the steady metabolism state [6] under the influence of enzyme activity [11, 12], enzyme concentration [11, 12], and energy expenditure [12, 18]. Nevertheless, when the strain is modified, the biosynthesis pathway is forced to express, and the combined constraints fail in biosynthesis efficiency simulation.

To simulate the biosynthesis efficiencies under forced pathway expression, PYF algorithm integrates the relationships between microbial biosynthesis tasks and growth interests by employing biosynthesis pathway expression degrees (Fig. 1a). This metric quantifies the growth disruption caused by forced expression of the product biosynthesis pathway, based on the decreased growth rate ($\overline{v_{bio}^{dec}}$) and the wild strain growth rate ($\overline{v_{bio}^{nfeg}}$) (Eqs. (1)–(2)). If the expression degree is low, the relationship between the biosynthesis task and the growth interest can be thought as a balanced relationship, otherwise, a conflictual relationship. By adding this metric to the lower bound constraint on mean growth rates, PYF captures the transition from conflict to harmony between biosynthesis and growth, allowing it to accurately simulate carbon source uptake processes and exchange metabolite release processes.

For production prediction in two-strain consortia, PYF employs mean-instantaneous flux mapping and numerical integration to enhance simulation accuracy. As depicted in Fig. 1b, the mean-instantaneous flux mapping constant is derived from the carbon source concentration curves of mono-cultures and inoculum ratios (Eqs. (12)–(13)). This constant facilitates the simulation of instantaneous fluxes in two-strain consortia based on the mean fluxes of mono-cultures (Eq. (14)), significantly reducing computational complexity. By dividing the co-culture time into small intervals and applying numerical iteration for integration, the algorithm calculates production levels over the entire co-culture duration (Eqs. (15)–(16)).

The integration of biosynthesis pathway expression degrees, mapping constants, and numerical integration enhances the adaptability of the PYF algorithm. This allows for accurate production simulations under diverse conditions, including variations in inoculum ratios, mono- and dual-carbon sources, single and multiple exchange metabolites, absence of enzyme catalytic parameters, and absence of mono-culture data. Consequently, the need for extensive data collection is significantly reduced.

Additionally, the PYF algorithm optimizes metabolic engineering strategies by performing sensitivity analysis on biosynthesis pathway expression degrees and mean-instantaneous flux mapping constants (Fig. 1b). This enables precise predictions of the effects of bottleneck reactions, gene expression levels, and carbon source utilization on production outcomes (Eqs. (17)–(18)). Unlike traditional methods, PYF does not require labor-intensive and specialized division and integration of polymicrobial metabolic networks, simplifying the simulation process.

### Scenario I: mono-metabolite exchange for hydroxytyrosol biosynthesis

#### Calculation of the optimal expression degrees

**Figure 2:**
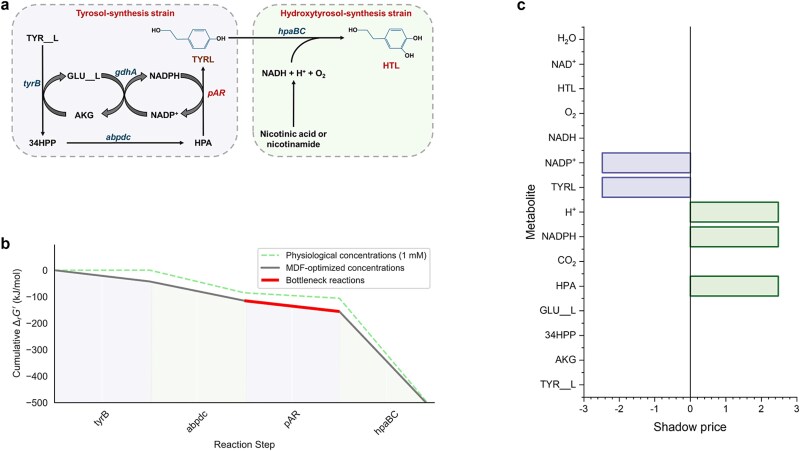
The tyrosol and hydroxytyrosol biosynthesis pathways and MDF thermodynamic analysis. (a) Pathways for tyrosol and hydroxytyrosol biosynthesis. (b) MDF thermodynamic drivers of reactions. (c) Shadow prices of metabolites. TYR__L: L__tyrosine, AKG: α-ketoglutarate, 34HPP: 4-hydroxyphenyl-pyruvate, GLU__L: L__glutamate, HPA: 4-hydroxyphenyl-acetaldehyde, TYRL: tyrosol, HTL: hydroxytyrosol.

To assess the accuracy of the PYF algorithm in predicting production within an *E. coli–E. coli* consortium, simulations were conducted on the hydroxytyrosol biosynthesis process, with tyrosol as a mono-exchange metabolite between the two strains [21] (Fig. 2a). FBA and thermodynamic constraints were incorporated to simulate the metabolic fluxes. The optimal expression degrees for tyrosol- and hydroxytyrosol-synthesis strains under these constraints are presented in Table 2, and the performance of each strain is shown in Appendix Table S1 (the same as below).

**Table 2:** The optimal expression degrees of the tyrosol biosynthesis pathway and the hydroxytyrosol biosynthesis pathway under different constraints

	Tyrosol-synthesis strain	Hydroxytyrosol-synthesis strain
FBA constraint	0.0011	0.0106
FBA and thermodynamic constraints	0.0005	0.0099

The expression degree reflects the growth disruption by forced expression of the product biosynthesis pathway. A higher expression degree corresponds to a more conflictual relationship between biosynthesis and growth, whereas a lower degree indicates a more balanced relationship. As shown in Table 2, the expression degrees for both tyrosol and hydroxytyrosol biosynthesis pathways are relatively low, suggesting a balanced relationship between biosynthesis tasks and growth interests. Notably, the optimal expression degrees under the combined FBA and thermodynamic constraints are lower than those obtained under FBA constraint alone. This difference likely arises from the low energy demands of the two pathways, where forced expression of the pathways requires minimal thermodynamic resources and has only a minor impact on strain growth.

#### MDF analysis of the optimal expression degrees

To confirm this hypothesis, MDF method [12] was employed to analyze the thermodynamic characteristics of the two biosynthesis pathways (Fig. 2b and 2c). From Fig. 2b, MDF analysis supports the simulated expression degrees of the biosynthesis pathways. The thermodynamic driving forces (the opposite value of ${\Delta }_r{G}^{\prime }$, Fig. 2b and Eq. (5), the same as below) for both exogenous pathways are substantial. Specifically, the bottleneck reaction of *pAR* and the MDF reached 40.71 kJ/mol, indicating high thermodynamic efficiency. This efficiency suggests that the target production can be achieved with relatively low expression degrees of the biosynthesis pathways from a thermodynamic perspective.

To further investigate the impact of metabolite concentrations on thermodynamic driving forces, we calculated the metabolite shadow prices for both pathways. A shadow price greater than zero indicates that increasing the concentration of the related metabolite promotes the pathway, while a negative value suggests the opposite effect. Figure 2c shows that, for the bottleneck reaction of *pAR*, the shadow prices of the substrates (4-hydroxyphenyl-acetaldehyde, H^+^, and NADPH) are greater than zero, and the products (tyrosol and NADP) are less than zero. This indicates that appropriately increasing the substrate concentrations and lowering the product concentrations could further improve the thermodynamic driving force and promote the two pathways.

#### Production prediction for hydroxytyrosol

**Figure 3:**
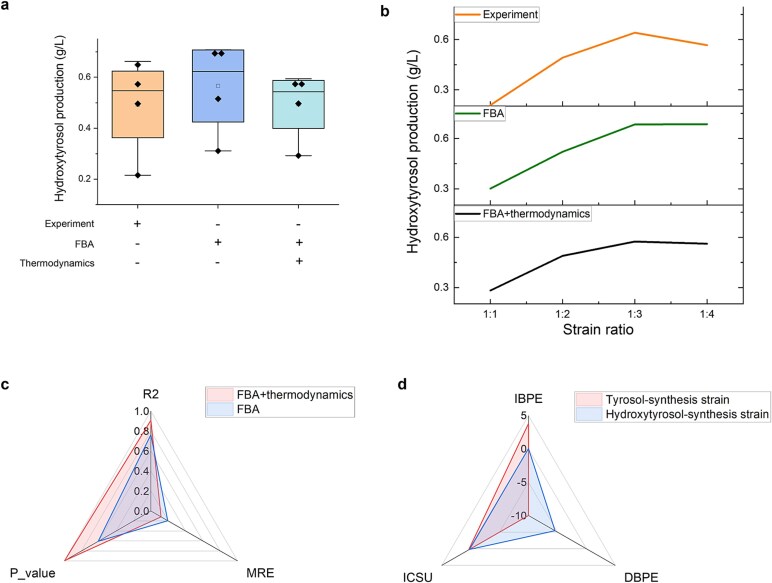
Hydroxytyrosol production over 6 h under different constraints and the change multiples of production under the unit changes of biosynthesis features. (a) The boxplot of productions. (b) The trend of productions with strain ratios of tyrosol-synthesis strain to hydroxytyrosol-synthesis strain. (c) The error and similarity of the simulated and the experimental productions. (d) The change multiples of production under the unit changes of biosynthesis features. IBPE: increasing biosynthesis pathway expression degrees, DBPE: decreasing biosynthesis pathway expression degrees, ICSU: increasing carbon source utilization.

To evaluate the accuracy of the PYF algorithm in predicting hydroxytyrosol production in the *E. coli–E. coli* consortium, the ratios between the growth lower bounds and the maximum growth rates were established based on optimal expression levels for the tyrosol- and hydroxytyrosol-synthesis strains, respectively. Simulations were performed over a 6-h co-culture period, iterating through 60 steps, under varying initial ratios of upstream to downstream strains (Fig. 3a and 3b, additional parameter details are shown in Appendix material S1, the same as below).

Figure 3a shows that, under combined FBA and thermodynamic constraints, the simulated hydroxytyrosol productions closely align with experimental results (the detailed values of productions were shown in Appendix Table S2, the same as below). The simulated trend in hydroxytyrosol production across different strain ratios and the simulated optimal strain ratio also aligns with experimental findings (Fig. 3b). Quantitatively, the mean relative error (MRE) between simulated and experimental values is 0.118, with a determination coefficient (*R*^2^) of 0.908 and a hypothesis test parameter that simulated values are similar to experimental values (*P* value) of 0.996 (Fig. 3c), reflecting strong predictive accuracy. In contrast, simulations with only the FBA constraint yield an MRE of 0.195, a lower *R*^2^ of 0.763, and a *P* value of 0.608, highlighting the critical role of thermodynamic constraints in enhancing prediction precision.

The thermodynamic constraint minimizes intracellular energy consumption through MDF value and promotes reactions to occur as spontaneously as possible. Without the thermodynamic constraint, the FBA-only model fails to capture the trend of hydroxytyrosol production relative to strain ratios, likely because FBA constrains fluxes solely under stoichiometric steady-state conditions without accounting for the intracellular environment effects on metabolism.

#### Metabolic engineering strategy optimization

To optimize the engineering strategy for hydroxytyrosol biosynthesis, a sensitivity analysis was conducted on the biosynthesis pathway expression degrees and mean-instantaneous flux mapping constants (the detailed values were calculated in Appendix Table S3, the same as below). The numerical difference approach was employed to evaluate the impact of unit changes in these parameters on hydroxytyrosol production, expressed as multiples of production increases or decreases under the strain ratio with the highest experimental production. Increasing the expression degrees can evaluate the effect of forced expression and pathway optimization on production, while decreasing the expression degree can assess the possibility of improving growth rate under the current production level. Furthermore, increasing the mean-instantaneous flux mapping constant can simulate the effect of enhanced carbon source utilization on production (same as below).

For the co-culture system, a relatively high hydroxytyrosol production has been experimentally achieved, with no reported cases surpassing these results. Therefore, optimization of the hydroxytyrosol biosynthesis system was performed solely through simulations. The results, shown in Fig. 3d, demonstrate that increasing the expression degree of the tyrosol-synthesis strain positively affects the production. Given the relatively low expression degree of this pathway (Table 2), significant improvements in hydroxytyrosol production could be achieved by forcing gene expression in the tyrosol biosynthesis pathway. Additionally, simulation results suggest that full utilization of glucose could modestly enhance hydroxytyrosol production. With the current glucose utilization rate at ~70%, there remains room for improvement in carbon source utilization efficiency. Therefore, it is feasible to increase the production by increasing the glucose utilization.

### Scenario II: dual-carbon sources for isobutyl-butyrate biosynthesis

#### Calculation of the optimal expression degrees

**Figure 4:**
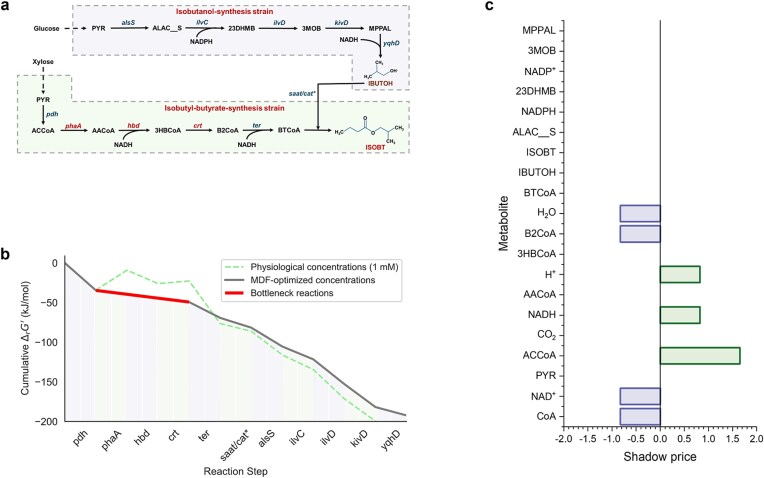
The isobutanol and isobutyl-butyrate biosynthesis pathway and MDF thermodynamic analysis. (a) Pathways for isobutanol and isobutyl-butyrate biosynthesis. (b) MDF thermodynamic drivers of reactions. (c) Shadow prices of metabolites. PYR: pyruvate, ACCoA: acetyl-CoA, AACoA: acetoacetyl-CoA, 3HBCoA: 3-hydroxybutyl-CoA, B2CoA: crotonyl-CoA, BTCoA: butyryl-CoA, IBUTOH: isobutanol, ISOBT: isobutyl-butyrate, ALAC__S: acetolactate, 23DHMB: 2,3-dihydroxy-isovalerate, 3MOB: 2-ketoisovalerate, MPPAL: isobutyraldehyde.

To assess the accuracy of the PYF algorithm in predicting production with co-utilized dual-carbon sources, simulations were conducted on the isobutyl-butyrate biosynthesis process, with glucose and xylose as co-utilized carbon sources [22] (Fig. 4a). FBA and thermodynamic constraints were incorporated to simulate the metabolic fluxes. Since the mono-culture experiment was not performed, two sets of co-culture data with the ratios of 1:4 and 1:9 for isobutanol and isobutyl-butyrate-synthesis strains were chosen to estimate the biosynthesis pathway expression degrees. After metabolic flux simulation of the co-culture data, the estimated optimal expression degrees for isobutanol and isobutyl-butyrate-synthesis strains under these constraints are presented in Table 3.

**Table 3:** The optimal expression degrees of the isobutanol-biosynthesis pathway and the isobutyl-butyrate biosynthesis pathway under different constraints

	Isobutanol-synthesis strain	Isobutyl-butyrate-synthesis strain
FBA constraint	0.0350	0.0110
FBA and thermodynamic constraints	0.0350	0.0110

As shown in Table 3, the expression degrees for both isobutanol and isobutyl-butyrate biosynthesis pathways are at low levels, suggesting a balanced relationship between biosynthesis tasks and growth interests. For both pathways, the optimal expression degrees under combined thermodynamic and FBA constraints are same as those under FBA constraint alone. This may indicate that the thermodynamic efficiencies of the pathways are in the medium range with no effect on the production.

#### MDF analysis of the optimal expression degrees

To confirm this hypothesis, MDF method [12] was carried out to analyze the thermodynamic characteristics of the two biosynthesis pathways (Fig. 4b and 4c). Figure 4b demonstrates that MDF analysis supports the simulated expression degrees of the biosynthesis pathways. The thermodynamic driving forces fall within a medium range, with the bottleneck reactions identified as *phaA*, *hbd*, and *crt*. The MDF value is 4.89 kJ/mol, indicating minimal influence on the biosynthesis pathway expression degrees.

Figure 4c further reveals key insights into the thermodynamics of the pathway. The shadow prices of the substrates (acetyl-CoA, H, and NADH) of reaction *phaA* are greater than zero, and the products (crotonyl-CoA, H_2_O, NAD, and CoA) of reaction *crt* are less than zero. This indicates that appropriately elevating the substrate concentration and decreasing the product concentration of the pathway from reaction *phaA* to reaction *crt* can further improve the thermodynamic driving force and promote the two biosynthesis pathways.

#### Production prediction for isobutyl-butyrate

**Figure 5:**
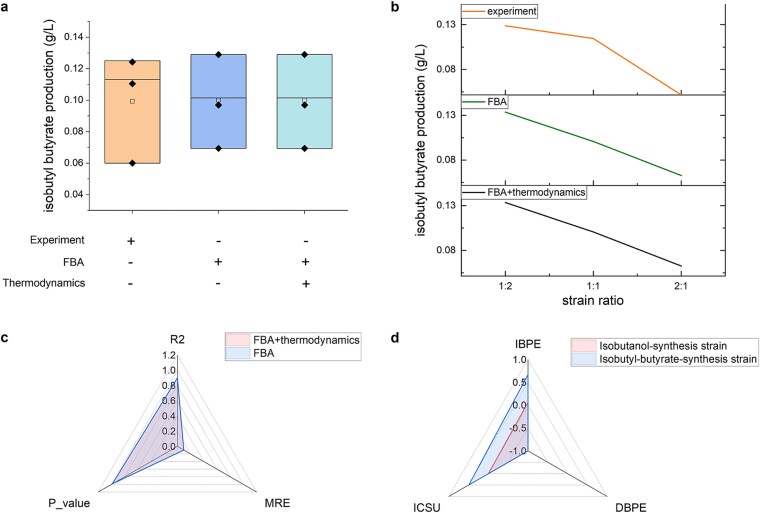
Isobutyl-butyrate production over 24 h under different constraints and the change multiples of production under the unit changes of biosynthesis features. (a) The boxplot of productions. (b) The trend of productions with strain ratios of isobutanol-synthesis strain to isobutyl-butyrate-synthesis strain. (c) The error and similarity of the simulated and the experimental productions. (d) The change multiples of production under the unit changes of biosynthesis features. IBPE: increasing biosynthesis pathway expression degrees, RBPE: decreasing biosynthesis pathway expression degrees, ICSU: increasing carbon source utilization.

As shown in Fig. 5a, the simulated isobutyl-butyrate production under combined FBA and thermodynamic constraints closely matches the production under FBA constraints alone, with both simulations aligning well with experimental results. Figure 5b demonstrates that the trend in isobutyl-butyrate production across different strain ratios and the predicted optimal strain ratio are consistent with experimental findings. Quantitatively, the MREs between simulated and experimental values are both 0.097, with an *R*^2^ of 0.900 and a *P* value of 0.984 (Fig. 5c) for both groups.

The negligible impact of thermodynamic constraint on isobutyl-butyrate production highlights that biosynthesis pathways with moderate thermodynamic efficiency impose minimal metabolic burdens. At this level, FBA constraint alone achieves high predictive accuracy. Moreover, PYF accurately infers biosynthesis pathway expression degrees using only two sets of co-culture data, enabling high prediction accuracy even in the absence of mono-culture data. This adaptability significantly reduces experimental costs.

#### Metabolic engineering strategy optimization

Figure 5d further illustrates the impacts of unit changes in these parameters on isobutyl-butyrate production. Enhancing the expression degree of the isobutyl-butyrate-synthesis strain strongly promotes the production, suggesting that optimizing the thermodynamic parameters of the isobutyl-butyrate biosynthesis pathway is critical. There are limited experimental studies on isobutyl-butyrate biosynthesis, but in a related pathway, Cong *et al*. increased butyl-butyrate production from 127.4 to 167.3 mg/L by elevating the expression of saat/cat* (a sub-thermodynamic bottleneck reaction predicted by MDF) in the butyl-butyrate-biosysthesis strain [23]. However, the main thermodynamic bottleneck reactions (*phaA*, *hbd*, and *crt*) predicted by MDF in isobutyl-butyrate biosynthesis remain underexplored. Optimizing these reactions, which convert acetyl-CoA to crotonyl-CoA, could significantly enhance isobutyl-butyrate production. Kang *et al*. achieved a 68% increase in butyl-butyrate production, an intermediate of isobutyl-butyrate biosynthesis, by optimizing acetyl-CoA supply [24]. Enhancing the expression degree of the isobutanol-synthesis strain has a slight positive effect on isobutyl-butyrate production. The *yqhD* reaction in the isobutanol-biosynthesis pathway has a low thermodynamic driving force (Fig. 4b), which limits pathway efficiency. This aligns with the findings of Beatriz *et al*., who increased isobutanol production by 19.3% by elevating *yqhD* gene expression [25].

Elevating the mean-instantaneous flux mapping constant of the isobutyl-butyrate-synthesis strain has a positive effect on the production, suggesting that increasing xylose utilization can enhance the biosynthesis. However, the current study found that xylose seemed to be difficult to be fully absorbed by *E. coli* [22]. Compared with the simulation results of biosynthesis pathway expression, the effective strategy to enhance the isobutyl-butyrate production should be biosynthesis pathway optimization rather than enhancement of xylose utilization.

### Scenario III: dual-metabolite exchange for *n*-butanol production

#### Calculation of the optimal expression degrees

**Figure 6:**
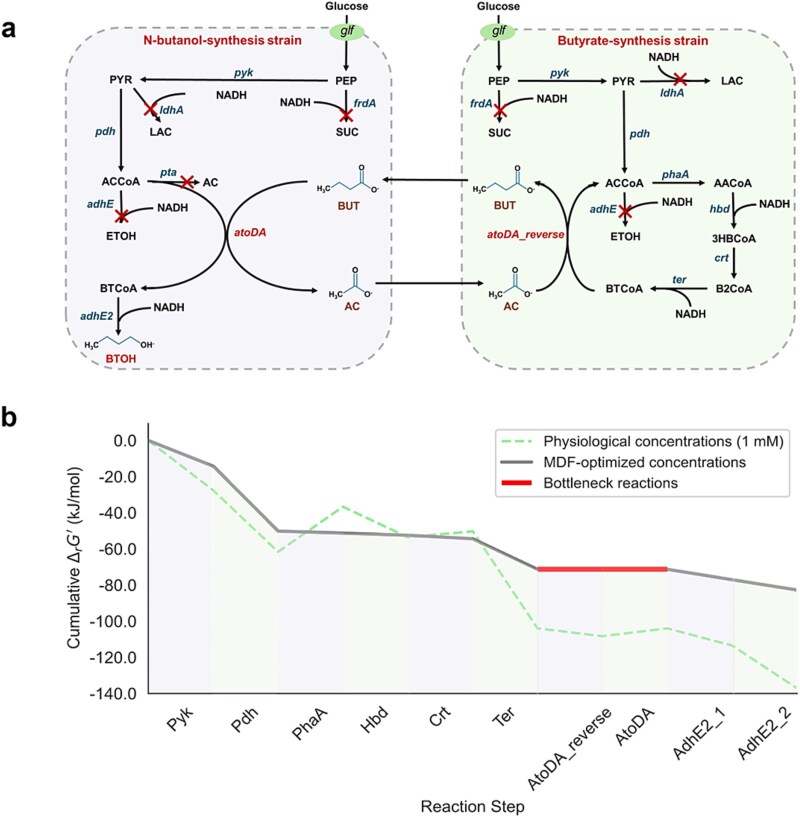
The butyrate and *n*-butanol biosynthesis pathway and MDF thermodynamic analysis. (a) Pathways for butyrate and *n*-butanol biosynthesis. (b) MDF thermodynamic drivers of reactions. PEP: phosphoenol-pyruvate, PYR: pyruvate, ACCoA: acetyl-CoA, BUT: butyrate, BTCoA: butyryl-CoA, AC: acetate, BTOH: *n*-butanol, AACoA: acetoacetyl-CoA, 3HBCoA: 3-hydroxybutyl-CoA, B2CoA: crotonyl-CoA.

To assess the accuracy of the PYF algorithm in predicting *n*-butanol production with dual-metabolite exchange, simulations were conducted on the *n*-butanol biosynthesis process, with butyrate and acetate as the dual-exchange metabolites between the two *E. coli* strains [26] (Fig. 6a). Because the enzyme turnover number (*k*_cat_ values) of the pathways are available, kinetic constraint was incorporated to further improve the simulation accuracy by accounting for enzyme concentration and catalytic efficiency. The metabolic fluxes were simulated under FBA, kinetic, and thermodynamic constraints. The optimal expression degrees for butyrate and *n*-butanol biosynthesis pathways under these constraints are presented in Table 4.

**Table 4:** The optimal expression degrees of the butyrate biosynthesis pathway and the *n*-butanol biosynthesis pathway under different constraints

	Butyrate-synthesis strain	*n*-Butanol-synthesis strain
FBA	0.6900	0.5000
FBA and kinetic constraints	0.5700	0.7500
FBA and thermodynamic constraints	0.7000	0.6600
FBA, kinetic, and thermodynamic constraints	0.5300	0.7600

As shown in Table 4, the expression degrees for both butyrate and *n*-butanol biosynthesis pathways are relatively high, indicating a conflictual relationship between biosynthesis tasks and growth interests. For the butyrate biosynthesis pathway, the optimal expression degree under combined FBA and kinetic constraints is lower than that observed under FBA constraint alone. This is likely due to the high catalytic efficiencies of the enzymes in this pathway (average *k_cat_* of ~6503.76/(g·h)) and the low enzyme requirement. The enforced pathway expression minimally occupies cellular enzyme resources, resulting in a mild slowdown of strain growth. From the kinetic perspective, target production can be achieved with a relatively low expression degree.

In contrast, the optimal expression degree for the *n*-butanol biosynthesis pathway under combined FBA and kinetic constraints is higher than that under FBA constraint alone. This is attributed to the pathway’s low catalytic efficiencies (average *k_cat_* of ~557.18/(g·h)) and high enzyme requirement. The enforced expression places a significant demand on cellular enzyme resources, leading to a marked slowdown in growth. From a kinetic perspective, achieving target production necessitates a relatively high degree of forced expression. When thermodynamic constraints are applied, the optimal expression degrees for both pathways increase relative to FBA constraints alone, highlighting the high energy requirements for pathway activity and the significant burden on cellular thermodynamic resources. Therefore, the slowdown of cell growth is great for each strain.

#### MDF analysis of the optimal expression degrees

To confirm the hypothesis about thermodynamic characteristics, MDF method [12] was carried out to analyze the two biosynthesis pathways (Fig. 6b). The analysis results support the simulated optimal expression degrees, revealing low thermodynamic driving forces for both pathways, with an overall MDF value of 0 kJ/mol. Bottleneck reactions, including *atoDA_reverse* and *atoDA*, exhibit driving forces of 5.99 and −5.99 kJ/mol, respectively, indicating low thermodynamic efficiency. This suggests that achieving target production requires high expression degrees of the biosynthesis pathways to compensate for the thermodynamic inefficiency from a thermodynamic perspective.

For the butyrate biosynthesis pathway, the optimal expression under combined FBA and thermodynamic constraints is slightly higher than that under FBA constraint alone, indicating approximately intermediate thermodynamic efficiency. This reflects that the forced expression of this pathway demanding only moderate energy supplementation from the cell. From a thermodynamic perspective, achieving target production requires slight increase of the expression degree. Interestingly, when both kinetic and thermodynamic constraints are applied together, the optimal expression degree for the butyrate biosynthesis pathway even decreases compared to combined FBA and kinetic constraints. This phenomenon may indicate that thermodynamic efficiency redistributes metabolic fluxes within the butyrate-synthesis strain, favoring reactions with high catalytic efficiencies and thereby reducing the need for forced expression.

In contrast, the *n*-butanol biosynthesis pathway exhibits a significantly higher optimal expression degree under combined FBA and thermodynamic constraints than under FBA alone, highlighting its low thermodynamic efficiencies. The pathway imposes substantial demands on cellular energy, significantly slowing growth. From a thermodynamic perspective, achieving target production requires a high forced expression degree. When all constraints (FBA, kinetic, and thermodynamic) are applied, the optimal expression degree becomes even greater, indicating high enzyme cost, energy requirement, and growth slowdown of *n*-butanol biosynthesis pathway. Therefore, achieving target production demands high forced expression degree.

The reversibility of the thermodynamic bottleneck reactions (*atoDA_reverse* and *atoDA*) further complicates pathway optimization, and results show that the shadow prices of the associated metabolites are zero. This implies that regulating intermediate metabolite concentrations is unlikely to effectively enhance the pathway. Therefore, improving production may require alternative strategies, such as optimizing enzyme activity or reallocating cellular resources through targeted metabolic engineering.

#### Production prediction for *n*-butanol

**Figure 7:**
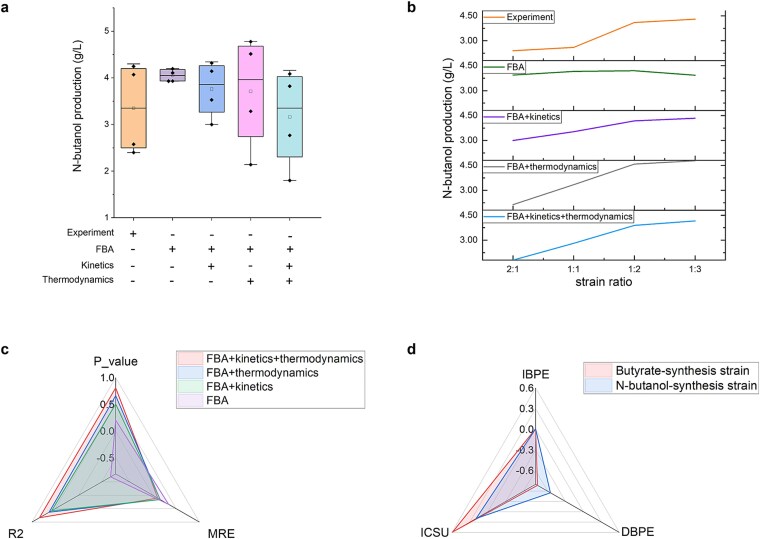
*n*-Butanol production over 24 h under different constraints and the change multiples of production under the unit changes of biosynthesis features. (a) The boxplot of productions. (b) The trend of productions with strain ratios of *n*-butanol-synthesis strain to butyrate-synthesis strain. (c) The error and similarity of the simulated and the experimental productions. (d) The change multiples of production under the unit changes of biosynthesis features. IBPE: increasing biosynthesis pathway expression degrees, DBPE: decreasing biosynthesis pathway expression degrees, ICSU: increasing carbon source utilization.

Figure 7a shows that, under combined FBA, thermodynamic, and kinetic constraints, the simulated *n*-butanol productions are close to the experimental results. The simulated production trends across different strain ratios and the predicted optimal strain ratio in *n*-butanol production also match the experimental observations (Fig. 7b). Quantitatively, the MRE between the simulated and experimental values is 0.104, with an *R*^2^ of 0.840 and a *P* value of 0.809 (Fig. 7c), reflecting strong predictive accuracy.

In comparison, simulations using only partial constraints showed reduced predictive accuracy. Under combined FBA and kinetic constraints, the MRE decreased to 0.159, *R*^2^ dropped to 0.579, and the *P* value was 0.505. Similarly, under combined FBA and thermodynamic constraints, the MRE was 0.155, *R*^2^ was 0.633, and the *P* value was 0.663. The model using FBA constraints alone failed to simulate the trend of *n*-butanol production relative to strain ratios, underscoring the importance of incorporating both kinetic and thermodynamic constraints. Kinetic constraints are crucial to simulate the cellular tendency to save enzyme costs, while thermodynamic constraints are essential for capturing the energy-saving behavior of cellular metabolic processes.

#### Metabolic engineering strategy optimization

To optimize the engineering strategy for *n*-butanol biosynthesis, a sensitivity analysis was conducted on the biosynthesis pathway expression degrees and mean-instantaneous flux mapping constants (Fig. 7d). From Fig. 7d, elevating the flux mapping constants significantly boosts *n*-butanol production, indicating that maximizing glucose utilization can greatly enhance biosynthesis efficiency. This finding aligns with experimental results from Yun-Peng *et al*., who improved *n*-butanol yield by ~42.2% in a system where glucose was nearly depleted compared to one with underutilized glucose [27].

In contrast, increasing the expression degrees of the biosynthesis pathways had only a slight positive effect on *n*-butanol production. This result reflects the influence of thermodynamic bottlenecks (*atoDA* and *atoDA_reverse*) in the pathways (Fig. 6b). These predicted bottleneck reactions are consistent with the findings of Yun-Peng *et al*., who enhanced the expression of genes related to these reactions and achieved a 20.7% increase in *n*-butanol yield compared to an unoptimized system [28]. These findings underscore the importance of both carbon source utilization and pathway optimization for improving *n*-butanol production.

### Comparison of PYF with other algorithms

To evaluate the performance of PYF comprehensively, it was compared with algorithms based on the “global optimum” assumption, the “Nash equilibrium” assumption, and the CulECpy algorithm, as summarized in Table 5. The results demonstrate that PYF achieves high simulation accuracy for *E. coli–E. coli* consortia, with an average MRE of 0.106 across three case studies. Compared with the latest flux simulation algorithm CulECpy, PYF reduces the MRE by ~61.6%. Furthermore, the *R*^2^ and *P* values for PYF simulations reach 0.883 and 0.928, respectively, indicating high model fitness and significantly enhancing the credibility of the simulation results.

**Table 5:** Comparison of the performance of PYF with other algorithms

Algorithms	Adapted strain numbers	MRE	*R* ^2^	*P* value	Advantages	Limitations	Runtime magnitudes	References
Algorithms based on “global optimum” assumption (joint-FBA, SteadyCom, OptCom, and cFBA)	≥2	N/A	N/A	N/A	Rapid metabolic flux simulation	Incompetence in competition state simulation	Second magnitude	[7, 8, 29, 30]
NECom	≥2	N/A	N/A	N/A	Metabolic flux simulation in competition states	Fail for simulation without a Nash equilibrium solution Complex mixed integer nonlinear programming process	Minute magnitude	[10]
CulECpy	≥2	0.275	−0.263	0.528	Multi-threaded computational setup Prediction of the optimal pathway division and optimal inoculum ratio	Complex division and integration of metabolic networks	Minute magnitude	[14]
PYF without kinetic constraint	2	0.119	0.823	0.755	No need for enzyme kinetic parameters Low cost of data collection	Incompetence in exploration of enzymic catalytic parameter influences	Minute magnitude	This study
PYF	2	0.103	0.889	0.799	Rapid exploration of biosynthesis–growth relationships and thermodynamic bottlenecks Rational production prediction and metabolic engineering strategy optimization	Dependence on the quality of metabolic networks	Minute magnitude^a^	This study

^a^The runtime magnitude refers to the maximum runtime required for all functions. For exploration of biosynthesis–growth relationship and thermodynamic bottleneck reactions, PYF requires second runtime magnitude. For production prediction and metabolic engineering strategy optimization, PYF requires minute runtime magnitude. N/A refers to not applicable.

Even in scenarios lacking *k_cat_* values, PYF demonstrates strong predictive capabilities. Using only FBA and thermodynamic constraints, it achieves an average MRE of 0.123, reducing the error by ~55.4% compared to CulECpy. Additionally, the *R*^2^ and *P* values improve to 0.814 and 0.879, respectively. This highlights PYF’s adaptability and its ability to accurately predict production without detailed enzyme catalytic data, thereby significantly reducing the data collection costs associated with enzyme characterization.

Beyond its predictive accuracy, PYF enables rational metabolic engineering strategy optimization through sensitivity analysis. It accurately predicts the effects of bottleneck reactions and carbon source utilization, providing actionable insights for improving metabolic efficiency.

Unlike other algorithms, PYF simplifies the simulation process by eliminating the need for labor-intensive and specialized division and integration of metabolic networks. Combining high accuracy with adaptability, PYF is a practical robust tool for simulating and optimizing metabolic processes in microbial consortia.

However, limited by the inaccuracy and incompleteness of the current coenzyme data, enzyme kinetic parameters, and thermodynamic information within metabolic networks, this study primarily focused on *E. coli–E. coli* consortia, where the metabolic models are relatively well established. To evaluate the broader applicability of PYF, we extended its application to other microbial consortia, such as *Corynebacterium glutamicum* and *Bacillus subtilis*. PYF successfully predicted production trends, optimal inoculation ratios, and effective metabolic engineering strategies (see Appendix Material S2 for details). These findings underscore the potential of PYF as a flexible and broadly applicable framework for microbial consortium modeling. It should be noted that the quantitative accuracy of its predictions remains contingent upon the quality and completeness of the underlying metabolic network models. With the progressive refinement of genome-scale metabolic reconstructions and the integration of more comprehensive biochemical parameters, the predictive fidelity and practical utility of PYF are expected to be significantly enhanced.

## Conclusions

This study presents a novel PYF algorithm for accurately predicting production in microbial consortia by integrating biosynthesis pathway expression degrees, flux balance analysis (FBA), enzyme kinetic, and thermodynamic constraints. PYF effectively addresses the relationships between biosynthesis and growth, allowing for accurate predictions across a wide range of scenarios, including mono-metabolite exchange, dual-carbon sources, and dual-metabolite exchange in *E. coli–E. coli* consortia. The algorithm demonstrated an average mean relative error (MRE) of 0.106, an *R*^2^ of 0.883, and a *P* value of 0.930, outperforming existing simulation methods by reducing the MRE by ~61.6%. In the absence of enzyme catalytic data, PYF obtained an average MRE of 0.123, reducing the error by ~55.4% and decreasing the data collection cost. Furthermore, the algorithm facilitates rational optimization of metabolic engineering strategies through sensitivity analysis, providing valuable insights into the effects of key metabolic parameters such as bottleneck reactions and carbon source utilization. By eliminating the need for complex metabolic network integration, PYF simplifies the simulation process and offers a scalable and efficient tool for production prediction and metabolic engineering strategy optimization.

**Key Points**

- Biosynthesis–growth relationships are effectively and rapidly explored.
- Productions of *E. coli*–*E. coli* consortia are accurately predicted.
- Metabolic engineering strategies are rationally optimized.

## Supplementary Material

Appendix_material_S1_bbaf295

Appendix_material_S2_bbaf295

Appendix_Table_S1_bbaf295

Appendix_Table_S2_bbaf295

Appendix_table_S3_bbaf295

Appendix_table_S4_bbaf295

## Data Availability

Data supporting the findings of this work are available within the paper and its Supplementary materials files. The codes of PYF are available at GitHub (https://github.com/ziyuanzu/PYF). The biosynthesis efficiency of each mono strain is shown in Appendix Table S1. The detailed values of productions are shown in Appendix Table S2. The biosynthesis pathway expression degrees and mean-instantaneous flux mapping constants are shown in Appendix Table S3. All the data sources are shown in Appendix Table S4. The simulation details of PYF are shown in Appendix material S1. The exploration in other biosynthesis consortia is shown in Appendix Material S2.
