# Supplementary material for: PYF: a multi-functional algorithm for predicting production and optimizing metabolic engineering strategy in Escherichia coli microbial consortia
Source: Brief Bioinform. 2025 Jun 21;26(3):bbaf295. doi: 10.1093/bib/bbaf295 (PMC12205937; doi:10.1093/bib/bbaf295)
Supplement: Appendix_material_S1_bbaf295 [file appendix_material_s1_bbaf295.docx]

**Parameter details of production simulation for *E. coli-E. coli* consortia**

For the mean-instantaneous flux mapping constant and initial settings of the hydroxytyrosol biosynthesis system, Since the hydroxytyrosol-biosynthesis strain was added after 9 h of mono-culture of tyrosol-biosynthesis strain, the initial tyrosol concentration in the co-culture stage was set to be 5.6 mM according to the tyrosol biosynthesis curve. The ratio of the initial concentration in the co-culture stage to the initial concentration in the mono-culture stage was approximately set to 5:1 for the tyrosol-biosynthesis strain according to the OD600 curve. Due to the lack of carbon source concentration curves, the mapping constant for tyrosol-biosynthesis strain was set according to the L-tyrosine concentration curve in mono-culture. The initial L-tyrosine concentration and the average L-tyrosine concentration was appropriately 10 mM and 6.5 mM separately, and the mapping constant for tyrosol-biosynthesis strain was thus about 1.54. The mapping constant for hydroxytyrosol-biosynthesis strain was set according to the tyrosol concentration curve in mono-culture. The initial tyrosol concentration and the average tyrosol concentration was appropriately 10 mM and 6 mM separately, and the mapping constant for hydroxytyrosol-biosynthesis strain was thus about 1.67.

For the mean-instantaneous flux mapping constant and initial settings of the isobutyl-butyrate biosynthesis system, Due to the lack of mono-culture experimental data, the glucose was assumed to be completely depleted throughout the process based on the experimental data of co-culture. Therefore, during the 96 h cultivation time, the average glucose concentration was approximated to be 1/2 (12.5 g/L) of the initial concentration (25 g/L). The mapping constant of the isobutanol-biosynthesis strain was calculated to be 2. Based on the experimental results that xylose-consuming strains begin to consume glucose as soon as the xylose concentration decreases to 0.6 g/L and the experimental data of the co-culture, the concentration of xylose at the initial moment was set to be 10 g/L, and the average concentration was set to be 5.3 g/L. The mapping constant of the isobutyl-butyrate-biosynthesis strain was calculated to be 1.88.

For the mean-instantaneous flux mapping constant and initial settings of the n-butanol biosynthesis system, According to the experimental result that the glucose was almost exhausted by butyrate-biosynthesis strain in monoculture system within 24 h, the glucose concentration at the initial moment was set to 12 g/L, the average glucose concentration within 24 h was 6 g/L, and mapping constants of the two strains were calculated to 2.
