# Supplementary material for: PYF: a multi-functional algorithm for predicting production and optimizing metabolic engineering strategy in Escherichia coli microbial consortia
Source: Brief Bioinform. 2025 Jun 21;26(3):bbaf295. doi: 10.1093/bib/bbaf295 (PMC12205937; doi:10.1093/bib/bbaf295)
Supplement: Appendix_material_S2_bbaf295 [file appendix_material_s2_bbaf295.docx]

**Exploration of PYF algorithm in other two-strain biosynthesis consortia**

To explore the simulation accuracy and adaptability of PYF algorithm in other two-strain biosynthesis consortia except *E. coli*, this research takes the fengycin biosynthesis consortium by *Corynebacterium glutamicum* and *Bacillus subtilis* as an example^[1]^. PYF explored the biosynthesis-growth relationship, simulated the fengycin production, and optimized the metabolic engineering strategies. In the consortia, both strains use sucrose as the carbon source, and *C. glutamicum* provided substrates for *B. subtilis* through secreting various amino acids such as proline, which in turn facilitated the fengycin production.

Since the metabolite names, upper reaction flux bounds and lower reaction flux bounds are not standardized, the *C. glutamicum* iCW773 model and the *B. subtilis* iYO844 model were unified. For the exchanged amino acids with different nomenclature, an amino acid name conversion module in the metabolic network model was set up, thus facilitating the coupling the metabolic fluxes of the two strains through the exchanged amino acids. According to the existing settings of iYO844 model, the ATP hydrolysis reaction fluxes of both models were set to 9 mM/h. For other reactions, the lower bounds of both models were set to 0 mM/h or -1000 mM/h, and the upper bounds were set to 0 mM/h or 1000 mM/h, according to the settings of the relatively mature *E. coli* iML1515 model.

The sucrose concentration was set to 29 mM according to the culture conditions of Gao et al. Since *B. subtilis* started to be inoculated 4 h after *C. glutamicum* was inoculated. The concentration of *C. glutamicum* at the beginning of co-culture stage was set to 11 times the inoculum according to the OD600 curve. It was assumed that the metabolite accumulation in pure culture was linearly correlated with the cultivation time. According to the accumulation after 72 h in pure culture of *C. glutamicum*, the proline concentration in the medium at the beginning of co-culture stage was set to 1.06 mM.

Since substrate concentrations except proline were not recorded, the fluxes of all amino acid secreting reactions can be used for fengycin biosynthesis in *C. glutamicum* iCW773 model were roughly set to 3 mM/h. Due to the lack of sucrose concentration change curves, sucrose was assumed to be completely absorbed and the average-instantaneous flux mapping constants for both strains were set to 2 approximately.

Currently, the models of *C. glutamicum* and *B. subtilis* suffer from the missing and wrong of key metabolites such as coenzymes and adenosine phosphate in the reactions, and the quality of calculated thermodynamic parameters such as Gibbs free energy is poor. Moreover, the enzymic kinetic parameters such as k_cat_ values are very inaccurate. Therefore, the consortium was investigated only by FBA constraint.

In the exploration of biosyntehsis-growth relationship, the calculated biosynthesis pathway expression degree of *C. glutamicum* was 0.459 and that of *B. subtilis* was 0.068. The high *C. glutamicum* biosynthesis pathway expression degree suggests that the relationship of biosynthesis task and growth interest are relatively conflict. This may be because of diverse amino acid synthesis tasks and high proline synthesis requirements. The low expression degree of *B. subtilis* biosynthesis pathway indicates a harmonious biosyntehsis-growth relationship. This may be because of the unique metabolic mode and ecological niche of *B. subtilis* developed in natural systems. *B. subtilis* ensure its survival by synthesizing and secreting antibiotics, such as fenzycin, to resist competition from eukaryotic strains and Gram-positive strains. However, due to missing and wrong of key metabolites, MDF thermodynamic analysis of the biosynthesis pathway was not done to verify the above hypothesis.

To predict production, PYF simulated the fenzycin production at different inoculum ratios (*C. glutamicum*:*B. subtilis*) of 1:1, 1:2, 1:3, 1:4 and 1:5, respectively, as shown in Figure. s1(a) and Figure. s1(b). The simulation results showed that the trends of fenzycin production with strain inoculum and the optimal inoculum ratio simulated by FBA were consistent with the experimental data. The errors in Fig. s1(c) showed that the Mean Relative Error (MSE) between the simulated and experimental values was 0.183, which was relatively low. P-value was 0.850, indicating slight difference. However, the determination coefficient (R^2^) was less than 0 due to the low variance of the experimental productions. These indicate that PYF performed well in predicting the trend of production changes and optimal inoculum ratios. The performance of PYF in quantitative prediction needs to be improved in the future when the metabolic networks are mature.

For the optimization of metabolic engineering strategies, this research calculated the effects of biosynthetic pathway expression and carbon source utilization on the fenzycin production using sensitivity analysis, as shown in Fig. s1(d). The simulation results showed that elevating the biosynthetic pathway expression degree of *B. subtilis* could greatly improve fenzycin production. Since the simulated relationship between biosynthesis tasks and growth interests of *B. subtilis* was harmonious, it is feasible to up-regulate the relevant genes to elevate the expression of the biosynthetic pathway. At the same time, elevating the biosynthetic pathway expression degree of *C. glutamicum* can weakly enhance fenzycin production. As the amino acid synthesis task of *C. glutamicum* is already burdensome, elevating its biosynthesis pathway expression degree is not meaningful. Since the carbon source is assumed already fully utilized, it is not possible to enhance the carbon source utilization of the strains. In conclusion, the most effective way to enhance fenzycin production is elevating the expression degree of *B. subtilis* biosynthetic pathway.


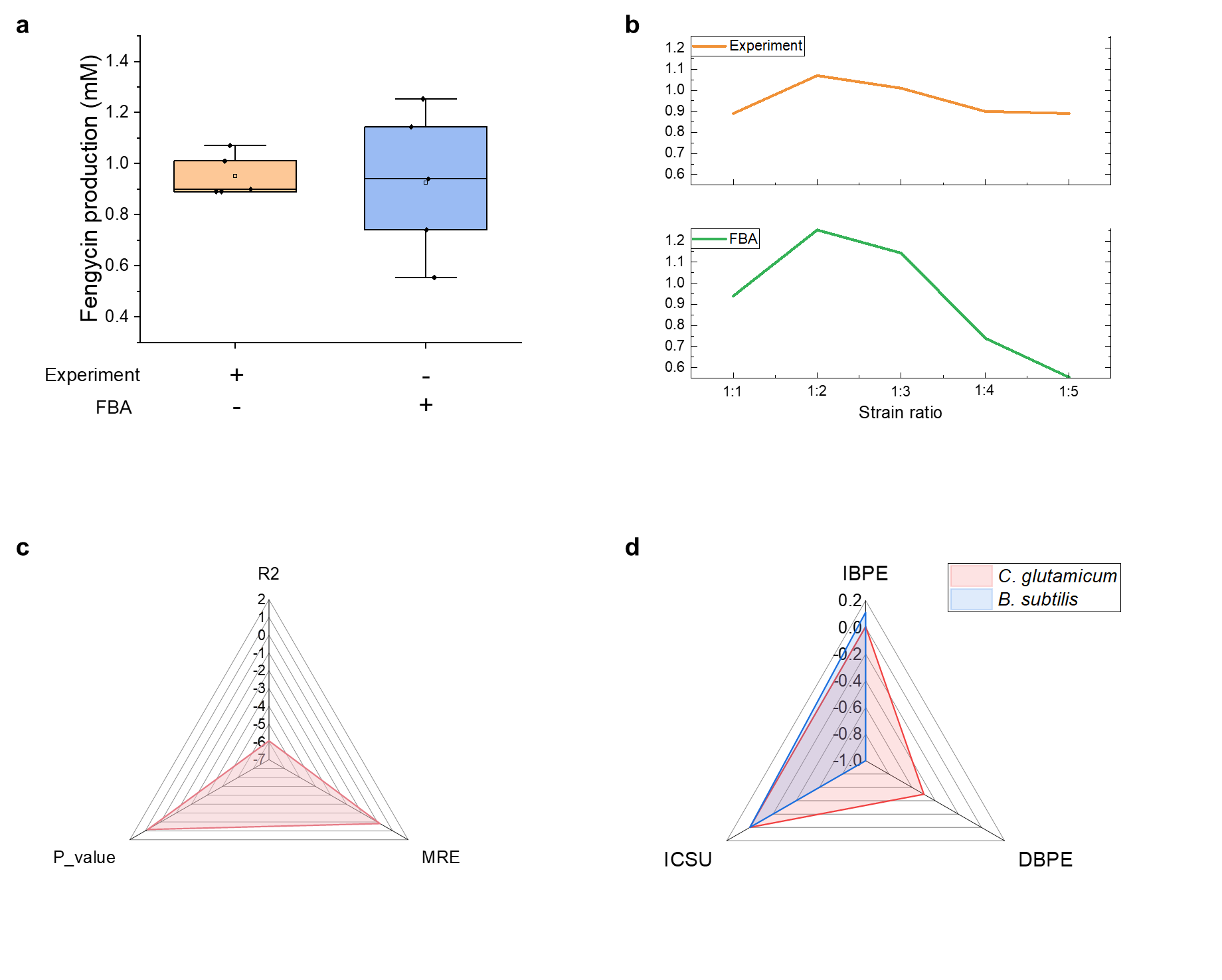


**Figure S1** **Fengycin production over 72 h under FBA constraint and** **the change multiples of production under the unit changes of biosynthesis features.** (a) The boxplot of productions. (b) The trend of productions with strain ratios of *C. glutamicum* to *B. subtilis*. (c) The error and similarity of the simulated and the experimental productions. (d) The change multiples of production under the unit changes of biosynthesis features. IBPE: increasing biosynthesis pathway expression degrees, DBPE: decreasing biosynthesis pathway expression degrees, ICSU: increasing carbon source utilization

**References**

[1] G.-R. Gao, S.-Y. Wei, M.-Z. Ding, Z.-J. Hou, D.-J. Wang, Q.-M. Xu, J.-S. Cheng, Y.-J. Yuan, Enhancing fengycin production in the co-culture of Bacillus subtilis and Corynebacterium glutamicum by engineering proline transporter, Bioresource Technology, 383 (2023) 129229.
