# Supplementary material for: PYF: a multi-functional algorithm for predicting production and optimizing metabolic engineering strategy in Escherichia coli microbial consortia
Source: Brief Bioinform. 2025 Jun 21;26(3):bbaf295. doi: 10.1093/bib/bbaf295 (PMC12205937; doi:10.1093/bib/bbaf295)
Supplement: Appendix_Table_S1_bbaf295 [file appendix_table_s1_bbaf295.docx]

Table S1 The biosynthesis efficiencies of mono-strains

| Biosynthesis consortium | Strain name | Target metalbolite | Biosynthesis efficiency |
| --- | --- | --- | --- |
| Hydroxytyrosol biosynthesis consortium | Tyrosol biosynthesis strain | Tyrosol | 6 mM |
|  | Hydroxytyrosol biosynthesis strain | Hydroxytyrosol | 7.99 mM |
| Isobutyl butyrate biosynthesis consortium | Isobutanol biosynthesis strain | Isobutanol | none record |
|  | Isobutyl butyrate biosynthesis strain | Isobutyl butyrate | none record |
| n-butanol biosynthesis consortium | Butyrate biosynthesis strain | Butyrate | 4.2, 5.6, 6.4 6.8 g/L at 2, 4, 6, 8 g/L acetate concentration, respectively |
|  | n-Butanol biosynthesis strain | n-Butanol | 1.9, 2.9, 3.7, 4.4 g/L at 3, 4, 5, 6 g/L butyrate concentration, respectively |
| Fengycin biosynthesis consortium | Proline biosynthesis strain | Proline and other precursor amino acids | 2.2 g/L for Proline and none record for other precursor amino acids |
|  | Fengycin biosynthesis strain | Fengycin | 0.52 g/L |
| If only one set of monoculture data is available, the biosynthesis efficiency is accurately simulated. If multiple sets of monoculture data are available, the optimal trend in biosynthesis efficiency is simulated | | | |
