# Supplementary material for: PYF: a multi-functional algorithm for predicting production and optimizing metabolic engineering strategy in Escherichia coli microbial consortia
Source: Brief Bioinform. 2025 Jun 21;26(3):bbaf295. doi: 10.1093/bib/bbaf295 (PMC12205937; doi:10.1093/bib/bbaf295)
Supplement: Appendix_Table_S2_bbaf295 [file appendix_table_s2_bbaf295.docx]

Table S2 The simulated and experimental productions in two-strain consortia

| Ratio of tyrosol-synthesis strain to hydroxytyrosol-synthesis strain | Experimental production (g/L) | Production (g/L) simulated by FBA constraint | Production (g/L) simulated by FBA and thermodynamic constraints |
| --- | --- | --- | --- |
| 1:1 | 0.216 | 0.311 | 0.292 |
| 1:2 | 0.508 | 0.538 | 0.506 |
| 1:3 | 0.662 | 0.707 | 0.594 |
| 1:4 | 0.585 | 0.708 | 0.581 |

| Ratio of isobutanol-synthesis strain to isobutyl-butyrate-synthesis strain | experimental production (g/L) | production (g/L) simulated by FBA constraint | production (g/L) simulated by FBA and thermodynamic constraints |
| --- | --- | --- | --- |
| 1:2 | 0.125 | 0.129 | 0.129 |
| 1:1 | 0.113 | 0.101 | 0.101 |
| 2:1 | 0.060 | 0.069 | 0.069 |

| Ratio of butyrate-synthesis strain to n-butanol-synthesis strain | Experimental production (g/L) | Production (g/L) simulated by FBA constraint | Production (g/L) simulated by FBA and kinetic constraints | Production (g/L) simulated by FBA and thermodynamic constraints | Production (g/L) simulated by FBA, kinetic and thermodynamic constraints |
| --- | --- | --- | --- | --- | --- |
| 2:1 | 2.40 | 3.94 | 3.00 | 2.14 | 1.80 |
| 1:1 | 2.60 | 4.16 | 3.53 | 3.34 | 2.81 |
| 1:2 | 4.10 | 4.20 | 4.18 | 4.58 | 3.89 |
| 1:3 | 4.30 | 3.93 | 4.34 | 4.78 | 4.16 |
