# Supplementary material for: PYF: a multi-functional algorithm for predicting production and optimizing metabolic engineering strategy in Escherichia coli microbial consortia
Source: Brief Bioinform. 2025 Jun 21;26(3):bbaf295. doi: 10.1093/bib/bbaf295 (PMC12205937; doi:10.1093/bib/bbaf295)
Supplement: Appendix_table_S3_bbaf295 [file appendix_table_s3_bbaf295.docx]

Table S3 The mean-instantaneous flux mapping constants of mono-strains

| Biosynthesis system | Biosynthesis strain | Mean-instantaneous flux mapping constant |
| --- | --- | --- |
| Hydroxytyrosol biosynthesis system | Tyrosol-biosynthesis strain | 1.54 |
|  | Hydroxytyrosol-biosynthesis strain | 1.67 |
| Isobutyl-butyrate biosynthesis system | Isobutanol-biosynthesis strain | 2 |
|  | isobutyl-butyrate-biosynthesis strain | 1.88 |
| N-butanol biosynthesis system | Butyrate-biosynthesis strain | 2 |
|  | N-butanol-biosynthesis strain | 2 |
