# Supplementary material for: PYF: a multi-functional algorithm for predicting production and optimizing metabolic engineering strategy in Escherichia coli microbial consortia
Source: Brief Bioinform. 2025 Jun 21;26(3):bbaf295. doi: 10.1093/bib/bbaf295 (PMC12205937; doi:10.1093/bib/bbaf295)
Supplement: Appendix_table_S4_bbaf295 [file appendix_table_s4_bbaf295.docx]

Table S4 The data sources of algorithm parameters and experimental data

|  | **Data name** | **Data source** |
| --- | --- | --- |
| **Algorithm parameter** | iML1515 model | Bigg models database (http://bigg.ucsd.edu/) |
|  | iCW773 model | Github website (https://github.com/notblank/sys-Bio-Cobra/tree/master) |
|  | iYO844 model | Bigg models database (http://bigg.ucsd.edu/) |
|  | Gibbs free energies of iML1515 reactions | Github website (https://github.com/tibbdc/ETGEMs/tree/master) for reactions in iML1515 model |
|  | Lower bounds and upper bounds of iML1515 metabolite concentrations | Github website (https://github.com/tibbdc/ETGEMs/tree/master) for metabolites in iML1515 model |
|  | Enzymic turnover numbers in iML1515 model | Github website (https://github.com/tibbdc/ETGEMs/tree/master) for enzymes in iML1515 model, Brenda database for other enzymes |
|  | Relative molecular weights of iML1515 enzymes | Github website (https://github.com/tibbdc/ETGEMs/tree/master) for enzymes in iML1515 model, Brenda database for other enzymes |
|  | Reaction equations in biosynthesis pathways | BioCyc database (https://www.biocyc.org/) |
| **Experimental data** | Experimental product productions of hydroxytyrosol | P. Gong, J. Tang, J. Wang, C. Wang, W. Chen, International Journal of Molecular Sciences, 24 (2023) 6944 |
|  | Strain ratios of the hydroxytyrosol biosynthesis consortium |  |
|  | Carbon source and substrate concentrations of the hydroxytyrosol biosynthesis consortium |  |
|  | OD600 curve of the hydroxytyrosol biosynthesis consortium |  |
|  | Experimental product productions of isobutyl butyrate | H. Seo, G. Castro, C.T. Trinh, ACS Synthetic Biology, 13 (2023) 259–268 |
|  | Strain ratios of the hydroxytyrosol isobutyl butyrate consortium |  |
|  | Carbon source and substrate concentrations of the isobutyl butyrate biosynthesis consortium |  |
|  | Experimental product productions of n-butanol | M. Saini, M. Hong Chen, C.J. Chiang, Y.P. Chao, Metabolic Engineering, 27 (2015) 76–82. |
|  | Strain ratios of the n-butanol biosynthesis consortium |  |
|  | Carbon source and substrate concentrations of the n-butanol biosynthesis consortium |  |
|  | Experimental product productions of fengycin | G.-R. Gao, S.-Y. Wei, M.-Z. Ding, Z.-J. Hou, D.-J. Wang, Q.-M. Xu, J.-S. Cheng, Y.-J. Yuan, Bioresource Technology, 383 (2023) 129229. |
|  | Strain ratios of the fengycin biosynthesis consortium |  |
|  | Carbon source and substrate concentrations of the fengycin biosynthesis consortium |  |
|  | OD600 curve of the fengycin biosynthesis consortium |  |
